# Unraveling the biochemistry and provenance of pupylation: a prokaryotic analog of ubiquitination

**DOI:** 10.1186/1745-6150-3-45

**Published:** 2008-11-03

**Authors:** Lakshminarayan M Iyer, AM Burroughs, L Aravind

**Affiliations:** 1 National Center for Biotechnology Information, National Library of Medicine, National Institutes of Health, Bethesda, MD 20894, USA; 2 Omics Science Center (OSC), RIKEN Yokohama Institute, 1-7-22 Suehiro-cho, Tsurumi-ku, Yokohama-shi, 230-0045 Kanagawa, Japan

## Abstract

Recently *Mycobacterium tuberculosis *was shown to possess a novel protein modification, in which a small protein Pup is conjugated to the epsilon-amino groups of lysines in target proteins. Analogous to ubiquitin modification in eukaryotes, this remarkable modification recruits proteins for degradation via archaeal-type proteasomes found in mycobacteria and allied actinobacteria. While a mycobacterial protein named PafA was found to be required for this conjugation reaction, its biochemical mechanism has not been elucidated. Using sensitive sequence profile comparison methods we establish that the PafA family proteins are related to the γ-glutamyl-cysteine synthetase and glutamine synthetase. Hence, we predict that PafA is the Pup ligase, which catalyzes the ATP-dependent ligation of the terminal γ-carboxylate of glutamate to lysines, similar to the above enzymes. We further discovered that an ortholog of the eukaryotic PAC2 (e.g. cg2106) is often present in the vicinity of the actinobacterial Pup-proteasome gene neighborhoods and is likely to represent the ancestral proteasomal chaperone. Pup-conjugation is sporadically present outside the actinobacteria in certain lineages, such as verrucomicrobia, nitrospirae, deltaproteobacteria and planctomycetes, and in the latter two lineages it might modify membrane proteins.

**Reviewers:**

This article was reviewed by M. Madan Babu and Andrei Osterman

## Introduction

It was recently shown that *Mycobacterium tuberculosis *contains a small protein, Pup (Rv2111c), that is covalently conjugated to the ε-NH_2 _groups of lysines on several target proteins (pupylation) such as the malonyl CoA acyl carrier protein (FabD) [1]. *Mycobacterium*, like most other actinobacteria, also possesses an archaeal-type proteasome that contains an AAA+ ATPase and two distinct NTN hydrolase-type peptidases [2]. Pupylation of FabD was shown to result in its recruitment to the mycobacterial proteasome and subsequent degradation analogous to eukaryotic ubiquitin-conjugated proteins. This remarkable conjugation reaction was found to be dependent upon another mycobacterial protein, the proteasome accessory factor (PafA) [1,3]. Unlike ubiquitin and related ubiquitin-like proteins (UBLs), which are conjugated to target lysines by means of successive trans-thiolation reactions involving their C-terminal glycine residue, Pup was shown to be conjugated via the γ-carboxylate of the terminal glutamate [1-3]. Based on this the discoverers of pupylation suggested that the conjugation process might involve a different biochemistry, but did not specify what this reaction might be [1].

Using sensitive sequence analysis methods we show that PafA, the protein required for pupylation, belongs to the glutamine synthetase fold and predict that it is likely to catalyze an ATP-dependent peptide ligase reaction.

## Results and discussion

### Phyletic patterns, genome organization and evolutionary relationships of Pup and PafA

**Figure 1:**
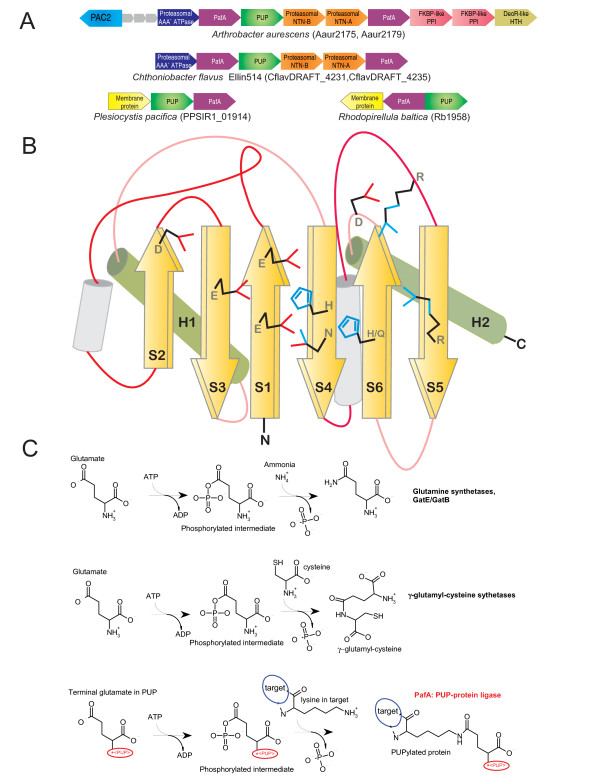
**PafA family gene neighborhoods, PafA topology diagram, and reactions catalyzed by PafA and related enzymes**. Conserved gene neighborhoods in (A) are depicted as arrows with the arrowhead pointing the 5' to 3' direction. The neighborhoods are labeled below with the name of a representative organism and the gene name corresponding to the PafA gene(s) from that neighborhood. The topology diagram in (B) depicts idealized conserved core secondary structural elements of the family. Elements that can be highly variable in the entire GS fold are shaded in gray. Conserved elements are labeled in the order in which they appear in the structure. Conserved residues contributing to catalysis are rendered as line drawings. The known and predicted reaction schemes for different members of the carboxylate-amine ligase superfamily are shown in (C). The member catalyzing the reaction is listed to the left of the reaction. In the case of GatB/GatE the reaction is catalyzed in situ on tRNA charged with a glutamate residue.

To understand better the pupylation process we investigated both Pup and PafA using sensitive sequence profile searches with the PSI-BLAST program and HMMer package. Pup was previously detected only in actinobacteria [1]. Our searches recovered Pup orthologs in all major actinobacteria lineages including the basal bifidobacteria and also sporadically in certain other bacterial lineages, such as nitrospirae, deltaproteobacteria (e.g. *Plesiocystis*), planctomycetes (e.g. *Rhodopirellula*) and the verrucomicrobia-chlamydia clade (e.g. *Methylacidiphilum*). The Pup proteins were all between 50–90 residues in length and a multiple alignment shows that they all contain a conserved motif with a G [EQ] signature at the C-terminus [Additional file 1]. Thus, all of them are suitable for conjugation via the terminal glutamate or the deamidated glutamine (as shown in the case of the *Mycobacterium *Pup [1]). The conserved globular core of Pup is predicted to form a bihelical unit with the extreme C-terminal 6–7 residues forming a tail in the extended conformation [Additional file 1]. Thus, Pup is structurally unrelated to the ubiquitin fold and has convergently evolved the function of protein modifier. Similar searches with the PafA protein of *Mycobacterium *showed that it had a phyletic pattern closely mirroring that of Pup; though in several lineages there were two paralogs of PafA (Fig. 1A and [Additional file 1]). PafA homologs (both, if two are present) and Pup are genomic neighbors in all bacterial lineages, with the Pup gene invariably being adjacent to one of the PafA genes (Fig. 1A). With the exception of the deltaproteobacterium *Plesiocystis *and the planctomycete *Rhodopirellula*, genes for the three proteasomal subunits are also associated with this conserved gene neighborhood (Fig. 1A). This suggests that in most currently available genomes with these genes there is a strong functional linkage between Pup, PafA and the archaeal-type proteasome, recapitulating the experimentally observed situation in *M. tuberculosis *[1,3].

**Figure 2:**
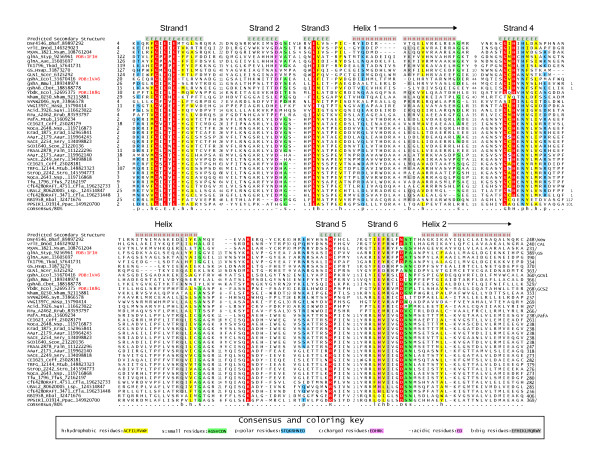
**Multiple alignment of PafA-like proteins and other members of the GS fold**. Proteins are labeled by gene name, organism abbreviation, and gi number, demarcated by underscores. Secondary structure assignments are given at the top of the alignment; E represents residues in β-strands while H represents residues in α-helices. Family names are listed to the right of the alignment, where new CAL is the new carboxylate-amine ligase of similar size as GCS2 mentioned in the text. Beyond the last helix shown in the alignment four additional conserved helices are predicted in PafA and are also found in the structures of other members of this superfamily. However, as these helices do not contribute to the active site and are poorly conserved in sequence we do not show them here. The residue coloring reflects at least 80% consensus conservation. Consensus similarity designations and coloring scheme are shown in the key. Absolutely conserved positions and residues essential for catalysis are shaded red. Organism abbreviations are as follows: Aae, *Aquifex aeolicus*; Aaur, *Arthrobacter aurescens*; Bmul, *Burkholderia multivorans*; Cbot, *Clostridium botulinum*; Ceff, *Corynebacterium efficiens*; Cfla, *Chthoniobacter flavus*; Dhaf, *Desulfitobacterium hafniense*; Dnod, *Dichelobacter nodosus*; Ecol, *Escherichia coli*; Faln, *Frankia alni*; Hasp, *Halobacterium *sp.; Hsap, *Homo sapiens*; Krad, *Kineococcus radiotolerans*; Lsp., *Leptospirillum *sp.; Mtub, *Mycobacterium tuberculosis*; Mxan, *Myxococcus xanthus*; Nham, *Nitrobacter hamburgensis*; Nsp., *Nocardioides *sp.; Ppac, *Plesiocystis pacifica*; Rbal, *Rhodopirellula baltica*; Rrub, *Rhodospirillum rubrum*; Scer, *Saccharomyces cerevisiae*; Scoe, *Streptomyces coelicolor*; Sery, *Saccharopolyspora erythraea*; Stro, *Salinispora tropica*; Styp, *Salmonella typhimurium*; Susi, *Solibacter usitatus*; Syn, *Synechococcus *sp.; Tfus, *Thermobifida fusca*; Tkod, *Thermococcus kodakarensis*.

PafA was earlier reported as a protein with no relationship to known protein domains [1,3]. A search with the *Saccharopolyspora *PafA homolog (SACE_2254; gi: 134098823) recovered γ-glutamyl-cysteine synthetase-2 (γ-glutamyl-cysteine ligase-2; GCS2) from *Saccharopolyspora *with borderline statistical significance (gi:134100361; expect-value = 0.08). Interestingly, this alignment completely spanned the GhExE signature (where 'h' is a hydrophobic residue and 'x' any residue), which is absolutely conserved in both PafA and the GCS2 families and forms part of the Mg^2+ ^and ATP binding active site of the latter enzymes (Fig. 1B and 2). To further explore the evolutionary affinities of the PafA family we prepared a multiple alignment and used an HMM derived from this alignment for an HHpred profile-profile comparison search against a library of HMMs derived from non-redundant PDB structures as seeds. This search recovered the GCS2 HMM (based on PDB: 1r8g) as the highly significant best hit (p-value= 10^-5^), with an alignment spanning the entire length of the GCS2 catalytic domain and matching all key conserved motifs (Fig. 2; see below). Thus, the PafA family appears to be a member of the glutamine synthetase (GS) fold to which GCS2 belongs [4,5]. While all known members of the GS fold catalyze ATP-dependent phosphotransfer reactions, they belong to either of two distantly related superfamilies: 1) The carboxylate-amine/ammonia ligases, which catalyze a two step ligase reaction involving phosphorylation of a carboxylate group (usually γ-carboxylate of glutamate) followed by ligation of the amino group of an amino acid (GCS1 and GCS2) or ammonia (glutamine synthetases) with the formation of an amide linkage (Fig. 1C) [6]. 2) The guanido kinases, which phosphorylate the guanido group of arginine or creatine [7,8]. Given that the GhExE is a distinctive signature only seen in the first superfamily, it became clear that PafA is a member of the carboxylate-amine/ammonia ligase superfamily.

To better understand the affinities of the PafA family within this superfamily and the functional implications of this relationship we first defined the conserved core shared by all carboxylate- amine/ammonia ligases using characterized structures. We generated a structural alignment of the glutamine synthetase, GatB and GatE proteins, which catalyze the *in situ *synthesis of glutamine or asparagine on Q-tRNA or N-tRNA charged with glutamate and aspartate respectively, and two families of γ-glutamyl-cysteine synthetases (GCS1 and GCS2) using the MUSTANG program. This alignment showed that despite several large family-specific inserts, the entire superfamily shared 6 conserved strands, typically in a 231465 arrangement, with at least two universally conserved helices occurring C-terminal to strands 3 and 6, respectively (Fig. 1B). These strands form a saddle-shaped structure with the active site located on the concave face and the conserved helices packing against the convex face. The structural alignment also revealed that the core strands 1, 2, 3, 4 and 6 contributed key catalytic residues to the active site in all members of this superfamily. The predicted secondary structure of the PafA family revealed the presence of equivalents of all conserved strands of this ligase superfamily (Fig. 1B, 2). Further, a comparison of motifs on equivalent strands showed that (Fig. 1B, 2): 1) the PafA family contains a GhExE on the core strand-1 which is equivalent to the Ex [EH] motif present in the first strand of all characterized superfamily members. 2) PafA shares with the rest of the superfamily conserved acidic residues on core strands 2 and 3, which are involved in contacting Mg^2+ ^and/or ATP. 3) In core strand-4 PafA contains a [HQ] x [NH] motif that is equivalent to the [HD] x [NH] motif that is present in all previously characterized members of this superfamily. This motif is critical for interacting with both the phosphate on the intermediate and a metal ion in the active site [6]. 4) In core strand-6 PafA displays a motif of the form [QH]×4D that corresponds to the motif Ex [RK]×2D seen in the equivalent strand of other members of the superfamily. The first conserved polar residue in this motif is located close to the active site metal and ATP. 5) Additionally, the PafA family shares with all carboxylate- amine/ammonia ligases, excluding the GatB and GatE families, a conserved arginine in core strand-5 and another arginine in the long loop N-terminal to this strand (Fig. 1B, 2). These arginines project into the active site surface and are likely to act as "arginine fingers" [9] in stabilizing the hyper-charged intermediate during phosphotransfer or participate in binding one of the substrates. Thus, the PafA family possesses all the features needed to function as an ATP-dependent carboxylate-amine ligase, like other members of this superfamily.

### Functional and evolutionary implications of PafA as a carboxylate-amine ligase

The above observation together with the experimental evidence and genomic context strongly imply that PafA is the Pup ligase, and catalyzes the ligation of the γ-carboxylate of the terminal glutamate (or glutamine deamidated to glutamate) of Pup to the ε-NH_2 _group of a lysine on the target protein (Fig. 1C). Many enzymes of the carboxylate-amine ligase superfamily, including GCS1 and GCS2, function as dimers. Hence, in light of the frequent presence of two PafA paralogs in most organisms, we propose that the Pup ligase is typically a heterodimer. However, in cases like *Mycobacterium*, with a single PafA gene, it is likely to be a homodimer. In several actinobacteria (e.g. *Arthrobacter, Streptomyces*) this gene neighborhood also includes two Fkbp-type peptidyl prolyl isomerases and a DeoR-family transcription factor (Fig. 1A). The former association suggests that prolyl isomerases might have an accessory role in pupylation of certain substrates. The associated DeoR transcription factor might regulate expression of the pupylation and protein degradation system by sensing a small molecule. Some actinobacterial Pup-proteasome gene neighbhorhoods contain another conserved protein typified by *Corynebacterium *cg2106 (PBD: 2p90), which is also found in archaea, frequently in the neighborhood of the proteasomal ATPase subunit. Most bacteria and archaea encode two cg2106 paralogs and sequence profile searches revealed that they are orthologs of the eukaryotic chaperone PAC2 required for proteasome assembly [10]. Cg2106 forms a trimeric torroid, suggesting that it might provide a scaffold for assembly of proteasomal peptidase subunits. As none of the other eukaryotic proteasomal chaperones have orthologs in archaea or bacteria, this protein is likely to represent the ancestral chaperone of the proteasome (Additional file 1). In both *Plesiocystis *and *Rhodopirellula*, we find no linkage between Pup/Pup ligase and genes for proteasomal subunits; instead they are linked to a gene for a membrane protein (Fig. 1A). Interestingly, these Pup ligases contain a remarkable insertion of 4 trans-membrane segments immediately C-terminal to the core strand-4 [Additional File 1]. Based on available structures of members of the GS fold these TM helices are predicted to stick out of the core fold without distorting it and are likely to anchor these Pup ligases to the cytoplasmic face of the cell membrane. Hence, in these organisms pupylation of membrane-associated proteins might have a regulatory role.

Given that the best hits for Pup ligases in profile-profile comparisons is the widely distributed GCS2 family, and the fact that the γ-glutamyl-cysteine synthetases catalyze a very similar reaction to pupylation, it is likely that the Pup ligase emerged in the actinobacterial lineage from a GCS2 precursor. We carried out multiple sequence profile searches with different starting points of carboxylate-amine/ammonia ligase superfamily to identify additional members. As a result we recovered two more previously uncharacterized families of these ligases [Additional file 1]. The first of these families is comprised of large proteins containing an N-terminal transglutaminase-like papain fold domain fused to a C-terminal domain of the carboxylate-amine/ammonia ligase superfamily (E.g. *Mycobacterium tuberculosis *Rv2566, gi: 15609703). Proteins of the second family (E.g. *Clostridium perfringens *CJD_1902, gi: 182624943) are similarly sized to GCS2 and are found in conserved gene neighborhoods encoding a glutamine amidotransferase-like thiol peptidase (in proteobacteria) or an Aig2-family γ-glutamyl cyclotransferase (in firmicutes) [11]. In neither of these cases small, conserved ORFs reminiscent of Pup are encoded in their gene neighborhoods. This observation, in conjunction with their domain fusions and gene-neighborhoods, suggests that they are likely to mediate peptide formation reactions in the context of synthesis of glutathione or related peptide secondary metabolites rather than conjugating proteins. Hence, pupylation appears to be a rather distinctive reaction, despite the shared biochemistry, that has emerged from a superfamily that otherwise specializes in cofactor (glutathione) or amino acid (glutamine) biosynthesis. In this respect it is reminiscent of the emergence of ubiquitination from precursors likewise involved in cofactor (molybdopterin and thiamine) and amino acid (cysteine) biosynthesis [12-14]. Thus, remarkably similarly covalent protein modifications by peptides or amino acids appear to have convergently evolved on at least 3 distinct occasions in unrelated folds of enzymes: 1) Ubiquitination in the Rossmanoid E1 fold and the distinct E2 fold [12]; 2) Pupylation in the GS fold and 3) Bacterial and eukaryotic N-end rule arginyl or leucyl ligation in the acetyltransferase fold [15].

## Materials and methods

Gene neighborhoods were determined using a custom script that uses completely sequenced genomes or whole genome shot gun sequences to derive a table of gene neighbors centered on a query gene. Then the BLASTCLUST program [16] is used to cluster products across the neighborhoods and establish conserved co-co-occurring genes. These conserved gene neighborhoods are then sorted as per a ranking scheme based on occurrence in at least one other phylogenetically distinct lineage ("phylum" in NCBI Taxonomy database), complete conservation in a particular lineage ("phylum") and physical closeness on the chromosome indicating sharing of regulatory -10 and -35 elements. Profile searches were conducted using the PSI-BLAST program with a default profile inclusion expectation (E) value threshold of 0.01 [17]. Profile-profile comparisons were performed using the HHpred program [18]. Multiple alignments were constructed using the Kalign program [19] followed by manual adjustments based on structural alignments generated using MUSTANG [20]. Protein secondary structure was predicted using a multiple alignment as the input for the JPRED program [21].

## Competing interests

The authors declare that they have no competing interests.

## Authors' contributions

LMI and LA were involved in the discovery process and writing the paper. AMB was involved in initiating interest in the project and preparing the alignments. All authors read and approved the final manuscript.

## Reviewer's comments

M. Madan Babu, MRC-LMB, University of Cambridge, Cambridge CB22QH, United Kingdom

In this manuscript, Lakshminarayan Iyer, Maxwell Burroughs and L Aravind report an important study that sheds light on the potential catalytic mechanism of how Pup, a small protein, gets post-translationally added to substrates. In Mycobacterium tuberculosis, it was recently shown that PafA was a factor that was important for such a modification to occur. Though this was known, the catalytic mechanism of how this is achieved remains unknown. In this work, using a combination of comparative genomic analysis, sequence and structure comparisons, the authors reveal that PafA is a distant evolutionary relative of the gamma-glutamyl-cysteine synthetase/glutamine synthetases. By a systematic comparison of available sequences of homologs and their structures, they identify critical residues that are important for function. Using these observations, the authors predict that PafA is likely to catalyze an ATP dependent ligation of the gamma-carboxylate of glutamate of Pup to lysines of the substrates.

They also show that Pup-conjugation is likely to be present sporadically outside actinobacteria.

In summary, this is an exciting and timely work, reporting a significant finding. Therefore, I would strongly support publication of this work in Biology Direct.

Questions

1. In instances where you do not find Pup proteins could it be a gene prediction error? Do you predict short ORFs that maybe missed by conventional gene prediction programs?

Authors' response: In all organisms where a PafA gene is found a gene encoding Pup is found as its neighboring gene. Being a small protein it has not been annotated in several actinobacteria and *Rhodopirellula*. We have translated some of these as examples and include it in additional file 1.

Andrei Osterman, Burnham Institute, La Jolla, CA, United States

The manuscript "Unraveling the biochemistry and provenance of pupylation: a prokaryotic analog of ubiquitination" by L. M. Iyer, A. M. Burroughs and L. Aravind conveys the most spectacular bioinformatics-based discovery. Everything about this short article is truly amazing, starting from its very modest size and as-a-matter-of-fact style of presenting a genuine intellectual breakthrough. Authors brilliantly combined comparative genomics, structural bioinformatics and biochemical reasoning to discover a novel enzymatic mechanism of tremendous biological importance. Although we are already quite spoiled by the scale of insightful functional inferences produced by bioinformatics and comparative genomics, this study constitutes a quantum leap into an entirely new dimension. The predicted mechanism is so obviously elegant that its "reduction to practice", which will inevitably follow very soon, will hardly add much to the story. In addition to establishing a new enzymology, the authors provided solid evidence that the physiological role of pupylation, at least in some bacteria, extends beyond tagging proteins to proteasomal cleansing. This observation opens a new line of studies that will likely follow. Finally, this paper provides an excellent tutorial in advanced bioinformatics and the most compelling illustration of its impact in biological discovery.

Questions

1. Do you believe that there is a specific terminal Gln deamidase working in TB? If yes, any candidates?

Authors' response: Gene neighborhoods do not reveal any candidates for glutamine deamidation. Given that the deamidation reaction is related to that proposed to be catalyzed by the Pup ligase it is possible that in cases where a terminal glutamine is found it first deamidates it before proceeding with the ligase reaction. Alternatively a non-specific amidase might be involved.

## Supplementary Material

Additional File 1 A complete list of conserved gene neighborhoods and comprehensive alignments of the PafA family, newly identified carboxylate-amine ligase families and PUP are provided. They can be accessed from: . Click here for file
