# Supplementary material for: Unraveling the biochemistry and provenance of pupylation: a prokaryotic analog of ubiquitination
Source: Biol Direct. 2008 Nov 3;3:45. doi: 10.1186/1745-6150-3-45 (PMC2588565; doi:10.1186/1745-6150-3-45)
Supplement: Additional File 1 — A complete list of conserved gene neighborhoods and comprehensive alignments of the PafA family, newly identified carboxylate-amine ligase families and PUP are provided. They can be accessed from: . [file 1745-6150-3-45-S1.html]

Unraveling the biochemistry and provenance of pupylation: a prokaryotic analog of ubiquitination
